# Analysis of SIRT1 Gene SNPs and Clinical Characteristics in Medication-Related Osteonecrosis of the Jaw

**DOI:** 10.3390/ijms25073646

**Published:** 2024-03-25

**Authors:** Bence Bojtor, Mihaly Vaszilko, Richard Armos, Balint Tobias, Janos Podani, Szofia Szentpeteri, Bernadett Balla, Balazs Lengyel, Henriett Piko, Anett Illes, Andras Kiss, Zsuzsanna Putz, Istvan Takacs, Janos P. Kosa, Peter Lakatos

**Affiliations:** 1 Department of Internal Medicine and Oncology, Semmelweis University, 1083 Budapest, Hungary; bojtorbence01@gmail.com (B.B.); armos.richard@semmelweis.hu (R.A.); tobias.balint@gmail.com (B.T.); lengyel.balazs.levente@semmelweis.hu (B.L.); piko.henriett@semmelweis.hu (H.P.); barkaszine.dr.illes.anett@semmelweis.hu (A.I.); putz.zsuzsanna@med.semmelweis-univ.hu (Z.P.); takacs.istvan@med.semmelweis-univ.hu (I.T.); kosa.janos@semmelweis.hu (J.P.K.); 2 Department of Oro-Maxillofacial Surgery and Stomatology, Semmelweis University, 1085 Budapest, Hungary; vaszilko.mihaly.tamas@semmelweis.hu (M.V.); szentpeteri.szofia.katalin@semmelweis.hu (S.S.); 3 Hungarian Research Network SE-ENDOMOLPAT Research Group, 1085 Budapest, Hungary; balladetti@gmail.com; 4 Department of Plant Systematics, Ecology and Theoretical Biology, Eötvös Loránd University, 1117 Budapest, Hungary; janos.podani@ttk.elte.hu

**Keywords:** SIRT1, rs932658, medication-related osteonecrosis of the jaw, single-nucleotide polymorphism

## Abstract

Certain genetic factors, including single-nucleotide polymorphisms (SNPs) in the *SIRT1* gene, have been linked to medication-related osteonecrosis of the jaw (MRONJ). This study examined four SNPs in the *SIRT1* gene and implemented multivariate statistical analysis to analyze genetic and clinical factors in MRONJ patients. Genomic DNA was isolated from peripheral blood samples of 63 patients of European origin treated for MRONJ, and four SNP genotypes in the gene encoding the SIRT-1 protein were determined by Sanger sequencing. The allele frequencies measured in the MRONJ population were compared with allele frequencies measured in the European population in the National Center for Biotechnology Information Allele Frequency Aggregator (NCBI ALFA) database. Genetic and clinical factors were examined with multivariate statistical analysis. A C:A allele distribution ratio of 77.8:22.2 was measured in the rs932658 SNP. In the ALFA project, a C:A allele distribution ratio of 59.9:40.1 was detected in the European population, which was found to be a significant difference (*p* = 4.5 × 10^−5^). Multivariate statistical analysis revealed a positive correlation (0.275) between the genotype of SNP rs932658 and the number of stages improved during appropriate MRONJ therapy. It is concluded that allele A in SNP rs932658 in the *SIRT1* gene acts as a protective factor in MRONJ.

## 1. Introduction

Medication-related osteonecrosis of the jaw (MRONJ) is a rare but serious side effect of mainly antiresorptive drugs, which significantly impairs the quality of life of affected patients [1]. MRONJ is defined as bone exposed or accessible through an extra or intraoral fistula in the maxillofacial region that has persisted for more than 8 weeks, current or previous treatment with antiresorptive agents alone or in combination with other drugs (e.g., antiangiogenic agents, immune modulators) and no history of metastatic disease or radiation exposure to the jaw [2]. Such antiresorptive agents include nitrogen-containing bisphosphonates and the receptor activator of nuclear factor kappa-Β ligand (RANKL) inhibitor drug denosumab. These antiresorptive drugs are effective in managing conditions, including osteoporosis and skeletal-related events (SREs), such as hypercalcemia of malignancy or pathological fractures due to bone metastasis of solid tumors (e.g., breast cancer, prostate cancer) or multiple myeloma [3,4]. MRONJ cases can be further classified into two distinct categories based on the assumed therapies. These are bisphosphonate-related osteonecrosis of the jaw (BRONJ) and non-bisphosphonate-related osteonecrosis of the jaw (non-BRONJ). Non-BRONJ encompasses instances induced by various pharmacological agents. These agents include denosumab, tyrosine kinase inhibitors (e.g., sunitinib, sorafenib or cabozantinib), everolimus (an mTOR-inhibitor), tocilizumab (an interleukin-6 receptor inhibitor) and anti-vascular endothelial growth factor (anti-VEGF) monoclonal antibodies, such as bevacizumab [5,6]. The prevalence of MRONJ ranges from 0 percent to 18 percent in cancer patients [7,8,9] and 0.02 percent to 0.3 percent in osteoporotic patients, depending on the antiresorptive agent used [10,11,12].

The exact pathomechanism of the disease is still unclear; however, several factors are thought to play a role in the development of MRONJ. Among others, the inhibition of bone remodeling, inflammation and infection are all possible key factors in the pathomechanism [13,14,15]. Over the last decade, a growing number of studies have suggested that genetic predisposition might also be a contributing factor to the development of MRONJ [16,17,18,19]. Several genes have been studied, including *CYP2C8* or *HERC4*, and although whole exome and genome sequencing studies and analysis of candidate genes have not led to the clear identification of the gene that causes MRONJ, research has focused attention on several promising genes. In this context, *SIRT1* stands out in view of its potential role in MRONJ development, although further studies are needed to determine its exact role in the process. A previous study suggested that the upregulation of the *SIRT1* gene might be a protective factor in the development of MRONJ [17].

The *SIRT1* gene encodes the Sirtuin-1 protein, which is a member of the sirtuins, a NAD+-dependent histone deacetylase protein family, involved in several crucial biological processes, such as inflammation, cell metabolism, aging or oxidative stress through the modulation of the activity of several transcription factors and enzymes [20,21]. *SIRT1* is also thought to be a key regulator in bone homeostasis via epigenetically regulating several genes involved in bone metabolism [22].

**Figure 1:**
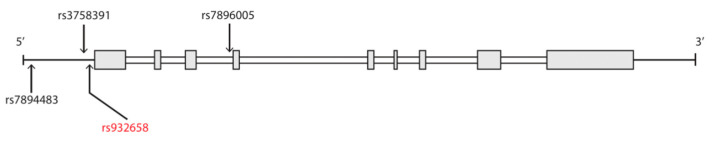
Schematic diagram showing the *SIRT1* gene with the SNPs tested. One single-nucleotide polymorphism (SNP) is located in the intronic region (rs7896005). One SNP is located in the 5′ upstream region (rs7894483). Two SNPs are located in the promoter region of the gene (rs932658, rs3758391). The distribution of alleles in the SNP highlighted in red is significantly different in the study population compared to the average population.

Based on previous whole exome sequencing studies, four single-nucleotide polymorphisms (SNPs) were examined in or in proximity to the *SIRT1* gene (rs932658, rs7896005, rs7894483 and rs3758391), which might have a causal role in the development of MRONJ. Figure 1 displays the schematic structure of the *SIRT-1* gene with the SNPs examined. Clinical data were also collected and evaluated. The aim of this study was to further understand the possible association between MRONJ and the aforementioned SNPs in the *SIRT1* gene in a single-center retrospective study.

## 2. Results

### 2.1. Study Population

The mean age of patients included in the study was 69.3 (range 45–91), 73% were female patients (n = 46), and 88.3% (n = 53) had a malignant disorder as primary disease, while 11.7% of patients (n = 7) suffered from osteoporosis as a reason for treatment. The majority of MRONJ cases (73.3%) were diagnosed as stage 2 disease (n = 44), 25% were deemed stage 3 (n = 15) and only one patient had stage 1 MRONJ. Descriptive statistics of the study population are shown in Table 1.

### 2.2. SIRT1 SNP Genotyping

**Figure 2:**
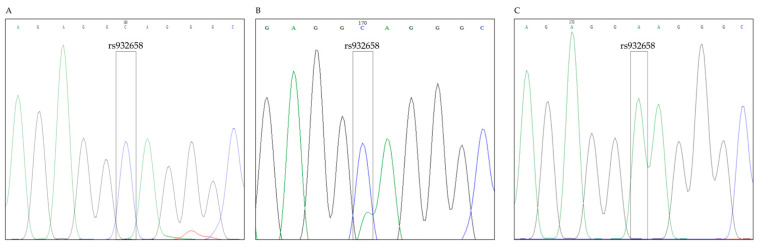
Representative Sanger sequencing diagrams of the rs932658 SNP. All sequencing diagrams were verified by its reverse base pair. (**A**) Sanger sequencing diagram of a homozygous C genotype. (**B**) Sanger sequencing diagram of a heterozygous CA genotype. (**C**) Sanger sequencing diagram of a homozygous A genotype.

There was a significant difference detected in the allele distribution of the rs932658 SNP by Chi-square test between the MRONJ group and the healthy European population measured in the National Center for Biotechnology Information Allele Frequency Aggregator (NCBI ALFA) database (*p* = 4.5 × 10^−5^) (Table 2). Representative Sanger’s sequence diagrams of SNP rs932658 are displayed in Figure 2.

**Figure 3:**
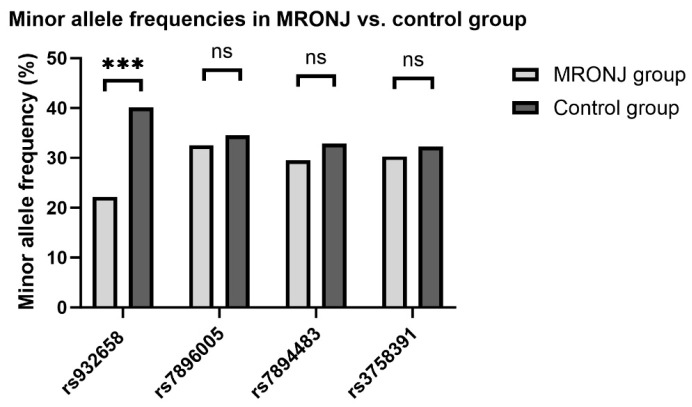
Bar chart showing the minor allele frequencies of the SNPs tested. The lighter color represents the Medication-Related Osteonecrosis of the Jaw (MRONJ) patients studied; the darker color represents the average population’s value based on the NCBI Allele Frequency Aggregator (ALFA) database. (https://www.ncbi.nlm.nih.gov/bioproject/PRJNA507278, accessed on 1 January 2024) A significant difference between MRONJ and the control group was detected in rs932658. *** *p* = 4.5 × 10^−5^. ns = not significant.

No further significant differences were seen in the other SNPs. Minor allele frequencies are displayed in Figure 3.

### 2.3. Multivariate Statistical Analysis

**Figure 4:**
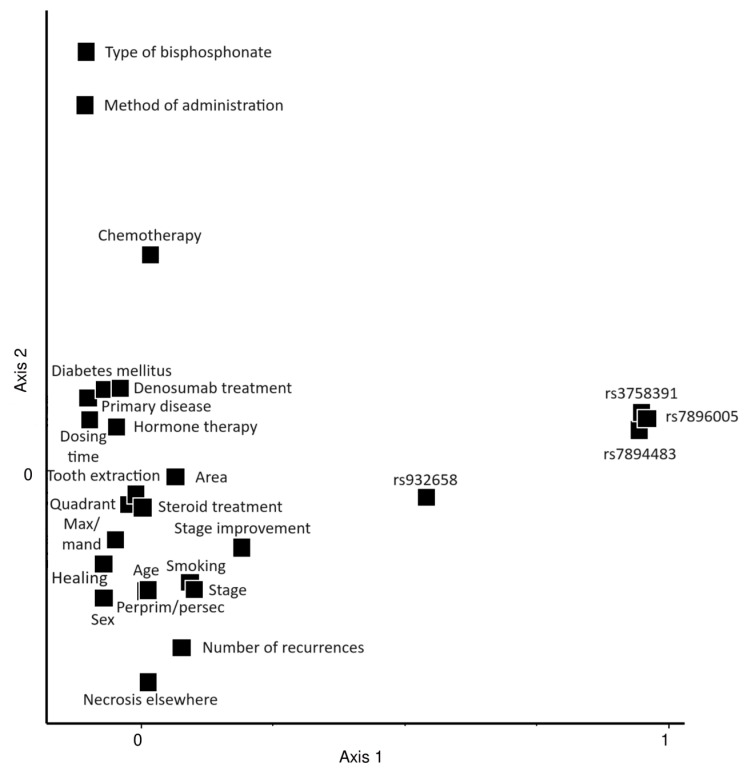
Principal components ordination of 26 variables used in the present study. Eigenvalues associated with axes 1 and 2 account for 12.8% and 8.5% of total variance, respectively. Necrosis elsewhere = osteonecrosis at a different site compared with the anatomical location of the primarily diagnosed MRONJ; Perprim/persec = per primam or per secundam wound healing after surgical treatment of MRONJ; Healing = complete healing after treatment (yes or no); Max/mand = maxillary or mandibular localization of MRONJ; Area = frontal, premolar or molar localization of MRONJ; Tooth extraction = prior tooth extraction in medical history (yes or no); Hormone therapy = prior hormone therapy in medical history (yes or no); Chemotherapy = prior chemotherapy in medical history (yes or no).

By multivariate statistical analysis of clinical and genetic variables, several strong correlations were found, which indicate the reliability of this novel statistical method. These include a high correlation (0.7063) between the type of bisphosphonate used and the method of bisphosphonate administration (e.g., oral, intravenous) or a relatively high positive correlation (0.2789) between the presence of chemotherapy and the type of bisphosphonate used. These relationships are expressed on the vertical principal component (PCA) axis (Figure 4). As a new finding, a relatively high positive correlation between the genotype of rs932658 and the number of stages improved after the appropriate non-surgical and surgical treatment was detected (0.275). This suggests that genetic factors, specifically the rs932658 SNP in the *SIRT1* gene, might play a role not only in the development of MRONJ but also in the clinical course of the disease. Patients with a more favorable genotype might be less likely to have MRONJ and have a higher rate of improvement once MRONJ has developed compared with less favorable genotype patients. As the diagram of Figure 4 shows, the other three genotypes are clustered; they are very highly correlated with one another (0.895 to 0.965). These appear uncorrelated with the other variables, thus explaining the contrast along the first PCA axis. A relatively high positive correlation (0.2417) was also detected between sex and healing. Previous dentoalveolar operation, corticosteroid therapy and diabetes mellitus did not show a correlation with other genetic or clinical variables. Smoking showed a positive correlation with the age of the patient (Supplement Table S2).

**Figure 5:**
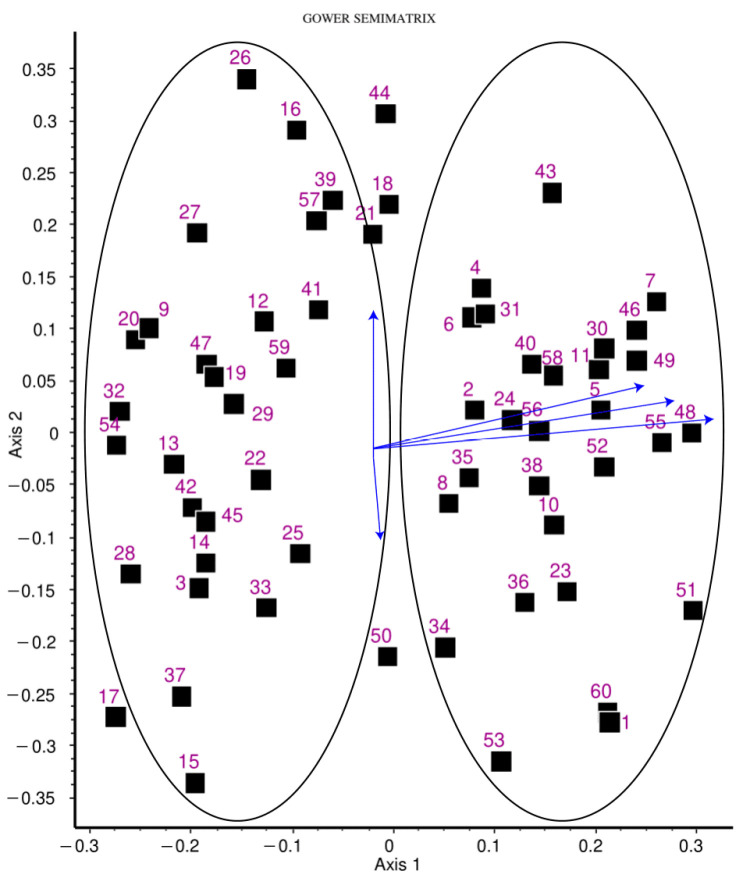
Principal coordinates ordination of 60 patients (full symbols). Eigenvalues associated with axes 1 and 2 account for 15% and 11% of total variance, respectively. Blue arrows and labels refer to variables with the highest correlations with either axis. Note that variable–axis correlation values were scaled down for best fit to the ordination, so that their relative positions and relative lengths matter only.

The PCoA of 60 patients is displayed in Figure 5 for axes 1 and 2. Apparently, the patients form two groups with a relatively large empty area separating them. To find the original variables best explaining this grouping, correlations between variables and the ordination scores for PCoA axes 1 and 2 were calculated. Then, variables with the highest correlations were selected. These were the three SNPs, rs7894483, rs7896005 and rs3758391, for axis 1 (r = 0.58, 0.61 and 0.59, respectively) and hormone therapy (r = 0.36) as well as the method of administration (r = −0.21) for axis 2. For visualization, the correlations were rescaled to best fit the ordination of patients, as usual in biplots, and arrows were drawn to point to their positions in Figure 5. As seen, the principal factors affecting the grouping of patients are the three genotypes, whereas none of the other clinical or genetic variables is influential.

## 3. Discussion

The findings of our study corroborated and validated the potential role of genetic factors, particularly SNPs associated with the *SIRT1* gene, as well as other clinical factors in the development of MRONJ in a Central European population, which is different from those originally investigated. Our research group has already utilized principal component analysis to evaluate genetic and clinical data in MRONJ patients more than a decade ago [18]. In that study, a strong positive correlation was detected between *CYP2C8* rs1934951 polymorphism and the anatomical localization of MRONJ. In an exome-wide association analysis of MRONJ patients with primary oncological disease, the strongest exome-level association was shown by the rs7896005 and rs375839 SNPs at the *SIRT1/HERC4* locus, which would be associated with a lower risk of MRONJ [16]. A genome-wide association study of five thousand bisphosphonate-treated European MRONJ samples revealed a strong association of the rs2736308 SNP on chromosome 8 with an increased risk of MRONJ. In silico analyses suggested that this polymorphism indirectly regulates SIRT1 expression [23]. Consistent with the results in this work, the *SIRT1* gene SNP rs932658 has also been demonstrated to be in association with MRONJ by regulating *SIRT1* expression [17]. Other research groups have suggested potential roles of SNPs in the *VEGF*, *CYP2C8*, *PPARG* and other genes in the development of MRONJ [19,24,25,26,27].

In this study a significant difference was detected in allele distribution for rs932658 between MRONJ patients and the NCBI Allele Frequency Aggregator (ALFA) (https://www.ncbi.nlm.nih.gov/bioproject/PRJNA507278, accessed on 1 January 2024) dataset subgroup measured in the European population, which suggests that individuals with certain genetic variations in the *SIRT1* gene may have a lower chance of developing MRONJ. Moreover, the relatively high positive correlation found between the genotype of rs932658 and the number of stages improved during the therapy of MRONJ that was found may suggest that individuals with more favorable genetic variations not only have less chance for developing MRONJ but also have a higher likelihood of healing. This is the first observation of this kind and raises the notion that genetic factors might also influence the response to therapeutic interventions in MRONJ. A previous study suggested that the minor allele (allele A) of rs932658 is associated with higher gene expression of the *SIRT1* gene, which might act as a protective factor in the development of MRONJ [17]. Our findings, in an independent Hungarian study cohort, confirm allele A in rs932658 as a potential protective factor in the development of MRONJ and predicts a greater degree of stage improvement in an already established MRONJ disease. This SNP, similar to other polymorphisms evaluated in this study (e.g., rs3758391), is located in the promoter region of the gene, which is universally understood to be crucial in the regulation of gene expression. One possible explanation of the functional importance of rs932658 might be its relative distance from the 5′-end of the *SIRT1* gene. However, further studies are called for to understand the mechanism of the effect of the rs932658 SNP on *SIRT1* gene expression.

Sirtuin proteins play an important role in bone biology. SIRT1 promotes bone formation by several molecular mechanisms. These include activating osteoblasts via stimulating the Wnt signaling pathway and decreasing the expression of sclerostin, an inhibitor of bone formation [28]. These findings established the start of several clinical trials investigating sirtuin-activating compounds for osteoporosis [28]. Despite significant progress over the past few years, many research questions have yet to be answered about sirtuin activators as potential therapeutic targets.

Consistent with the data in the literature [29], our study suggests that the prevalence of MRONJ is higher in women than in men. This is likely due to the higher prevalence of the primary diseases treated with antiresorptive agents in the female population (e.g., breast cancer, osteoporosis). However, the relatively high positive correlation between the sex of the patient and healing suggests that the patient’s sex might not be irrelevant in the clinical course of MRONJ. In our study group, female patients also had a higher tendency to heal from MRONJ than male patients. Between the sex of the patient and the genotype of rs932658, there was no positive correlation detected, so there might be factors other than genetic factors determining the correlation between the sex of the patient and healing. Other previously described risk factors like corticosteroid therapy or diabetes mellitus did not show a correlation with other clinical or genetic variables in our study.

Principal coordinates analysis revealed distinct patient groupings, primarily influenced by the genotypes of the three SNPs, i.e., rs7894483, rs7896005 and rs3758391, respectively (r = 0.58, 0.61, 0.59). The patients in the distinct groups might act differently in some clinical respects, so further studies may be needed to identify the relevance of this apparent patient grouping.

In discussing the outcomes of our investigation, it is imperative to acknowledge the advantages that underpin the significance of the findings presented in this article. This study was conducted on a relatively large and independent cohort of Central European MRONJ patients. This enabled us to detect genetic associations with greater reliability. Furthermore, a novel multivariate statistical analysis was deployed to assess the possible correlations between clinical and genetic variables. The results of the multivariate statistical analysis further validated the findings made by genotyping. This innovative methodological approach not only adds knowledge to current discourse but also might set a precedent for further investigations in this field.

It is also crucial to acknowledge the limitations of this study. The sample size of sixty-three patients, while providing valuable insights, may not fully capture the diversity of genetic variations that could contribute to MRONJ. Additionally, the study was based on a slightly incomplete dataset, and further research with larger and more diverse cohorts is required to validate and extend these findings.

The findings presented here might be useful in the future due to multiple reasons. Firstly, the possible role of SIRT1 in MRONJ and, more broadly, in bone biology was further validated by our findings. The association of MRONJ with the identified SNP implicates the important role of sirtuins in the pathomechanism of this adverse drug reaction, resulting in a severe decrease in the quality of life of affected patients. These results suggest that the modulation of sirtuins by pharmacological agents (e.g., STACs) might present a promising new field of therapeutics in MRONJ. Moreover, with the improvement of knowledge on the genetic background of MRONJ, new risk stratification tools based on the genetic predisposition of the patient can be developed, further aiding personalized medicine. Furthermore, these SNPs can be determined relatively easily and cost-effectively and might help patients avoid MRONJ, which could ease the load on caregivers and help patients and doctors concentrate on the management of the often malignant primary disease.

There is still a definite need for further research regarding the wide genetic landscape of MRONJ and, specifically, the role of the *SIRT1* gene in the disease. Since genetic factors may vary slightly in the investigated population, further validation of the association between rs932658 and the risk of MRONJ should be conducted in populations with different genetic backgrounds.

In summary, this research provides insights into the genetic and clinical factors underlying MRONJ, thus promoting a better understanding of its development and progression. The introduction of novel multivariate statistical analysis might also contribute to increasing our knowledge of MRONJ. There are some MRONJ risk assessment questionnaires published in the literature [30,31], whereas no prevalent, internationally accepted and validated a priori risk estimation algorithm for MRONJ has been available. In addition, there is absolutely no procedure that takes any genetic data into account in the assessment. The potential integration of genetic data into clinical management offers the possibility of personalized treatment in the future. A future goal would be to screen out high-risk patients for whom antiresorptive therapy should or should not be considered. Contribution to that goal with the results of this study is intended.

## 4. Materials and Methods

### 4.1. Patients

Sixty-three consecutive patients of Hungarian origin suffering from MRONJ were enrolled in this study. All MRONJ patients were treated at the Department of Oro-Maxillofacial Surgery and Stomatology, Semmelweis University. After detailed medical history taking, physical and laboratory examination, peripheral blood samples were taken from the patients. For three of them, the collection of previous medical data was unsuccessful; therefore, these patients were excluded from multivariate data analysis. All patients were informed and asked for consent before enrolling them in this study. The 1964 Declaration of Helsinki and its subsequent amendments were followed in this study. The study had the approval of the institutional ethics committee of Semmelweis University of Budapest (Semmelweis University Regional and Institutional Committee of Science and Research Ethics, protocol code: 10862-1/2016/EKU; IV/4613-4/2020/EKU). Access to human data was restricted to members of the research team. A password-protected medium was used to store the data. The authors confirm that all the methods were carried out in accordance with the applicable guidelines and regulations.

### 4.2. Genotyping

Four SNPs in the *SIRT1* gene were genotyped in the 63 subjects. Peripheral blood samples were collected, and genomic DNA was extracted from each subject using High Pure PCR Template Purification kit (Roche Diagnostics, GmbH, Mannheim, Germany). The PCR mixture (20 μL) contained 1 μL genomic DNA (50 ng/μL), 2 × 0.50 μL primer, 10 μL of JumpStart Taq Readymix (SIGMA-ALDRICH, Co., 3050 Spruce Street, St. Louis, MO 63103 USA) and 8 μL ultrapure PCR water. Cycling conditions comprised an initial cycle at 94 °C for 5 min followed by 40 cycles of 94 °C for 30 s, 60 °C for 30 s and by a final step at 72 °C for 1 min. Sanger dideoxy sequencing was performed to determine the genotype of the SNPs (Eurofins Genomics Europe GmbH, Ebersberg, Germany).

### 4.3. Statistical Analysis

The distribution of *SIRT1* SNP genotypes and alleles in the NCBI Allele Frequency Aggregator (ALFA) reference database (https://www.ncbi.nlm.nih.gov/bioproject/PRJNA507278, accessed on 1 January 2024) and MRONJ populations was analyzed by Chi-square tests. Statistics with a *p*-value < 0.05 were considered significant. Chi-square tests were performed using IBM SPSS 28.0 software (SPSS Inc., Chicago, IL, USA).

For further analysis, the data were summarized in a matrix with 60 rows (patients) and 26 columns (variables) (Supplement Table S1). Conventional multivariate techniques cannot be applied to this dataset for two reasons. The variables are not homogeneous regarding the measurement scale. A few are ratio-scale variables (age, duration of treatment and number of recurrences) for which arithmetic differences between values are meaningful. For the ordinal variables (stage, stage improvement), only the sequence of possible states can be interpreted. The majority of variables are nominal; that is, the only information that can be obtained by comparing two values is their equality or non-equality. Examples are sex (male or female), primary disease (lung cancer, osteoporosis, etc.) or smoking (yes or no). Also, the SNP genotypes are nominal, each value corresponding to a combination of alleles. For example, for *SIRT1* rs932658, 1 means CC, 2 means AC and 3 means AA in position. Furthermore, the data are incomplete; 138 data points (8.5%) are unknown due to various factors (lack of measurement, etc.). In such cases, to reveal data structure with patients or variables as units of comparison, special methods are called for. Objects may be compared using the Gower dissimilarity index [32], whereas correlations among variables of the mixed type may be calculated by the d-correlation formula recently suggested by Podani et al. (2023) [33]. Reducing dimensionality, thus revealing underlying data structure, is then made available through principal coordinates analysis (PCoA) of Gower dissimilarities and principal components analysis (PCA) from the correlation matrix. The results of both ordination procedures are displayed by scatter diagrams with axes as dimensions, most commonly selecting the first two. The relative importance of these dimensions is expressed by percentage contributions of eigenvalues to the total variance. The larger the sum of the eigenvalues for the axes shown, the more faithful the two-dimensional representation with respect to the entire data space. Calculations were performed by the DCORR application [33] and the SYN-TAX package [34].

## Figures and Tables

**Table 1:** Summary of the clinical characteristics of the study population.

Characteristics	MRONJ Patients n = 63
Age (years) (mean ± SD) *	69.33 ± 11.06
Sex	
Female	46
Male	17
Duration of treatment (months) (mean ± SD) *	46.42 ± 39.77
Disorder *	
Breast cancer	26
Prostate cancer	11
Myeloma multiplex	11
Osteoporosis	7
Lung cancer	2
Melanoma malignum	1
Colon cancer	1
Cervical cancer	1
Chemotherapy *	
Yes	38
No	22
Hormone deprivation therapy	
Yes	32
No	28
Antiresorptive agent *^,^ **	
Alendronate (p.o.)	1
Ibandronate (p.o.)	4
Risedronate (p.o.)	1
Zoledronate (iv./inj.)	29
Denosumab (inj.)	29
MRONJ localization *^,^ ***	
Maxilla	19
Mandibula	42
MRONJ stage *^,^ ****	
1	1
2	44
3	15

* There were no available clinical data for three patients. ** Four patients had both bisphosphonate and denosumab. *** One patient had both maxillar and mandibular involvement. **** Based on the American Association of Oral and Maxillofacial Surgeons’(AAOMS) Position Paper on Medication-Related Osteonecrosis of the Jaws—2022 update.

**Table 2:** Summary of the genotypes and allele frequencies. NCBI ALFA: National Center for Biotechnology Information Allele Frequency Aggregator for European population. *p*-value is for the statistical difference between the study and European populations.

SNP	Genotype 1	Genotype 2	Genotype 3	Allele Frequency	ALFA	*p*-Value
rs932658	CC: 65.1% (n = 41)	CA: 25.4% (n = 16)	AA: 9.5% (n = 6)	C:A 77.8:22.2	C:A 59.9:40.1	*p* = 4.5 × 10^−5^
rs7896005 *	GG: 47.4% (n = 27)	GA: 40.3% (n = 23)	AA: 12.3% (n = 7)	G:A 67.5:32.5	G:A 65.5:34.5	ns. (*p* = 0.64)
rs7894483 **	AA: 9.9% (n = 6)	AT: 39.3% (n = 24)	TT: 50.8% (n = 31)	A:T 29.5:70.5	A:T 32.9:67.1	ns. (*p* = 0.43)
rs3758391 **	CC: 50.8% (n = 31)	CT: 37.7% (n = 23)	TT: 11.5% (n = 7)	C:T 69.7:30.3	C:T 67.7:32.3	ns. (*p* = 0.64)

* Genotyping of six samples was unsuccessful. ** Genotyping of two samples was unsuccessful. ns. = not significant.

## Data Availability

Original data generated and analyzed during this study are included in this published article or in the data repositories listed in References.

## Supplementary Materials

The following supporting information can be downloaded at: https://www.mdpi.com/article/10.3390/ijms25073646/s1.
