# Supplementary material for: Analysis of SIRT1 Gene SNPs and Clinical Characteristics in Medication-Related Osteonecrosis of the Jaw
Source: Int J Mol Sci. 2024 Mar 25;25(7):3646. doi: 10.3390/ijms25073646 (PMC11011248; doi:10.3390/ijms25073646)
Supplement: Supplementary file 1 [file ijms-25-03646-s001.zip › Suppl2.pdf]

		Sex	Age	Prim.d.	DiabMel	Chemoter	Steroid	Deno.	Bisph.	Horm.ther.	Me.Adm.	T.adm.	Dent.op.	Smok	Loc.	Stag.	Necros.	Stage imp.	Heal.	Rec.	932658	7896005	7894483	3758391	PerP	Quadrant	Area
Sex	ROW 1	1.0000	.1115	.3859	-.0268	.0541	.0042	-.0129	-.1514	-.0151	-.0718	-.0207	-.0196	-.0963	.0626	.0168	.1746	-.0188	.2417	.0564	-.0444	-.0196	-.0329	-.0417	-.0159	.0177	-.0605
Age	ROW 2	.1115	1.0000	.0598	-.0566	.0357	.0174	.0181	-.0925	-.0047	-.0744	.1769	.0051	.3137	.0379	.0218	.0566	-.0266	.1091	.0187	.0103	.0117	-.0059	-.0043	-.0032	.0002	.0122
Primary disease	ROW 3	.3859	.0598	1.0000	-.0019	.1767	-.0005	.0407	.0809	.2935	.0382	.1155	-.0036	-.0419	-.0163	.0074	-.0045	-.0328	.0376	-.0796	-.0721	-.0298	-.0280	-.0397	-.0956	.0296	-.0086
DiabMelitus	ROW 4	-.0268	-.0566	-.0019	1.0000	.0003	-.0286	-.0079	.0414	.0292	.0570	.0870	-.0179	-.1112	-.0696	-.0012	-.0750	-.0479	.0508	-.0836	-.0303	-.0305	-.0274	-.0308	.0771	-.0441	-.0802
Chemotherapy	ROW 5	.0541	.0357	.1767	.0003	1.0000	.1105	-.0141	.2800	.0155	.2237	-.0293	-.0129	-.0484	-.0133	-.0381	-.0597	.0292	.0271	-.0282	.0115	.0335	.0423	.0392	-.0577	.0084	-.0172
Steroid treatment	ROW 6	.0042	.0174	-.0005	-.0286	.1105	1.0000	-.0162	-.0101	-.0175	-.0246	-.0314	-.0179	-.0116	-.0153	.0042	.0576	.0391	.0825	-.0121	-.0078	-.0014	.0003	-.0043	.1649	.0132	-.0069
Denosumab	ROW 7	-.0129	.0181	.0407	-.0079	-.0141	-.0162	1.0000	-.0110	.0804	.2628	.0078	-.0024	.0079	.0004	-.0018	.0079	-.0146	.0382	-.0117	.0042	-.0151	-.0206	-.0153	-.0064	.0087	.0050
Type of bisphos.	ROW 8	-.1514	-.0925	.0809	.0414	.2800	-.0101	-.0110	1.0000	.0195	.6287	.0074	-.0119	-.0853	.0127	-.1514	-.1505	-.1718	-.1362	-.1360	-.0776	-.0171	-.0153	-.0273	-.0854	-.0061	.0251
Hormone ther.	ROW 9	-.0151	-.0047	.2935	.0292	.0155	-.0175	.0804	.0195	1.0000	-.0117	.0443	.0384	-.0110	-.0168	-.0016	-.0149	.0215	-.0136	-.0406	-.0156	-.0247	-.0204	-.0121	.0253	.0244	-.0016
Meth. of adm.	ROW 10	-.0718	-.0744	.0382	.0570	.2237	-.0246	.2628	.6287	-.0117	1.0000	.0239	-.0197	-.0279	.0361	-.0656	-.0620	-.0400	.0718	-.0388	-.0942	-.0502	-.0215	.0046	-.0351	.0063	-.0855
Treatment time	ROW 11	-.0207	.1769	.1155	.0870	-.0293	-.0314	.0078	.0074	.0443	.0239	1.0000	-.0110	-.0925	.0235	.0523	.0187	.0718	-.0546	-.2965	-.1145	-.0413	-.0445	-.0328	-.0242	-.0366	-.0174
Dentoalv. op.	ROW 12	-.0196	.0051	-.0036	-.0179	-.0129	-.0179	-.0024	-.0119	.0384	-.0197	-.0110	1.0000	.0351	-.0222	-.0191	-.0161	.0003	.0427	.0162	-.0186	-.0182	.0023	-.0025	.0448	-.0141	-.0152
Smoking	ROW 13	-.0963	.3137	-.0419	-.1112	-.0484	-.0116	.0079	-.0853	-.0110	-.0279	-.0925	.0351	1.0000	.0526	-.0186	.0610	-.0479	-.1136	.1290	.0868	.0616	.0168	.0317	-.0403	.0558	-.0146
Loc.(max/mand)	ROW 14	.0626	.0379	-.0163	-.0696	-.0133	-.0153	.0004	.0127	-.0168	.0361	.0235	-.0222	.0526	1.0000	-.0626	.2598	-.0345	-.0679	.0297	-.0555	-.0259	-.0094	-.0283	-.0134	.5729	.0379
Stage	ROW 15	.0168	.0218	.0074	-.0012	-.0381	.0042	-.0018	-.1514	-.0016	-.0656	.0523	-.0191	-.0186	-.0626	1.0000	-.0186	.3905	.0188	.0982	.0861	.0323	.0434	.0016	-.0854	-.0221	-.0652
Necrosis elsewh.	ROW 16	.1746	.0566	-.0045	-.0750	-.0597	.0576	.0079	-.1505	-.0149	-.0620	.0187	-.0161	.0610	.2598	-.0186	1.0000	-.0184	.0945	.2737	-.0148	.0139	.0196	-.0074	.0771	.1254	-.0362
Stage improv.	ROW 17	-.0188	-.0266	-.0328	-.0479	.0292	.0391	-.0146	-.1718	.0215	-.0400	.0718	.0003	-.0479	-.0345	.3905	-.0184	1.0000	.1532	-.3251	.2750	.0778	.0733	.0848	.1284	-.0170	.0513
Healing	ROW 18	.2417	.1091	.0376	.0508	.0271	.0825	.0382	-.1362	-.0136	.0718	-.0546	.0427	-.1136	-.0679	.0188	.0945	.1532	1.0000	-.0408	-.0900	-.0447	-.0480	-.0201	.1704	-.0355	-.0925
Numb. of recurr.	ROW 19	.0564	.0187	-.0796	-.0836	-.0282	-.0121	-.0117	-.1360	-.0406	-.0388	-.2965	.0162	.1290	.0297	.0982	.2737	-.3251	-.0408	1.0000	-.0833	.0640	.0625	.0624	.1815	.0003	-.0237
S-1_932658	ROW 20	-.0444	.0103	-.0721	-.0303	.0115	-.0078	.0042	-.0776	-.0156	-.0942	-.1145	-.0186	.0868	-.0555	.0861	-.0148	.2750	-.0900	-.0833	1.0000	.3787	.3494	.3518	-.0189	-.0217	.0764
S-1_7896005	ROW 21	-.0196	.0117	-.0298	-.0305	.0335	-.0014	-.0151	-.0171	-.0247	-.0502	-.0413	-.0182	.0616	-.0259	.0323	.0139	.0778	-.0447	.0640	.3787	1.0000	.9292	.8939	-.0052	-.0108	.0440
S-1_7894483	ROW 22	-.0329	-.0059	-.0280	-.0274	.0423	.0003	-.0206	-.0153	-.0204	-.0215	-.0445	.0023	.0168	-.0094	.0434	.0196	.0733	-.0480	.0625	.3494	.9292	1.0000	.9651	-.0013	-.0076	.0597
S-1_3758391	ROW 23	-.0417	-.0043	-.0397	-.0308	.0392	-.0043	-.0153	-.0273	-.0121	.0046	-.0328	-.0025	.0317	-.0283	.0016	-.0074	.0848	-.0201	.0624	.3518	.8939	.9651	1.0000	.0046	-.0238	.0432
PerPrim/PerSec	ROW 24	-.0159	-.0032	-.0956	.0771	-.0577	.1649	-.0064	-.0854	.0253	-.0351	-.0242	.0448	-.0403	-.0134	-.0854	.0771	.1284	.1704	.1815	-.0189	-.0052	-.0013	.0046	1.0000	-.0277	.0618
Quadrant	ROW 25	.0177	.0002	.0296	-.0441	.0084	.0132	.0087	-.0061	.0244	.0063	-.0366	-.0141	.0558	.5729	-.0221	.1254	-.0170	-.0355	.0003	-.0217	-.0108	-.0076	-.0238	-.0277	.0000	.0622
Area	ROW 26	-.0605	.0122	-.0086	-.0802	-.0172	-.0069	.0050	.0251	-.0016	-.0855	-.0174	-.0152	-.0146	.0379	-.0652	-.0362	.0513	-.0925	-.0237	.0764	.0440	.0597	.0432	.0618	.0622	.0000
